# Protective Roles of Thymoquinone Nanoformulations: Potential Nanonutraceuticals in Human Diseases

**DOI:** 10.3390/nu10101369

**Published:** 2018-09-25

**Authors:** Ali H. El-Far, Soad K. Al Jaouni, Weikun Li, Shaker A. Mousa

**Affiliations:** 1 Department of Biochemistry, Faculty of Veterinary Medicine, Damanhour University, Damanhour 22511, Egypt; ali.elfar@damanhour.edu.eg; 2 Department of Hematology/Pediatric Oncology, Faculty of Medicine, King Abdulaziz University, Yousef Abdulatif Jameel scientific chair of Prophetic Medicine Application, Faculty of Medicine, King Abdulaziz University, Jeddah 21589, Saudi Arabia; saljaouni@kau.edu.sa; 3 The Pharmaceutical Research Institute, Albany College of Pharmacy and Health Sciences, Rensselaer, NY 12144, USA; weikun.li@acphs.edu

**Keywords:** thymoquinone, nanoformulation, biological activities, human diseases control

## Abstract

The focus on nanotechnology for improved bioavailability and drug delivery is of increasing importance for control of different human diseases. Therefore, numerous nanoformulations have been developed for the oral bioavailability of different drugs. This review introduces applications of nanomedicine to enhance the biological activities of thymoquinone (TQ) to control different diseases in several in vivo studies as a preliminary investigation for human disease treatment with nano-TQ. Nano-TQ effectively augments the anticancer roles of doxorubicin by upregulation of P53 and downregulation of Bcl2 and potentiates paclitaxel’s apoptosis in MCF-7 breast cancer cells. Moreover, nano-TQ protects against diabetes, inflammation, CNS, and hepatotoxicity, mainly by enhancement of organs’ antioxidant status. We summarize the pros and cons of several FDA approved nanoparticle-based therapeutics and discuss the roadblocks in clinical translation, along with potential nano-TQ strategies to overcome these roadblocks. From this review, we can conclude that nano-TQ may be considered as a promising nutraceutical for human health.

## 1. Introduction

**Figure 1:**
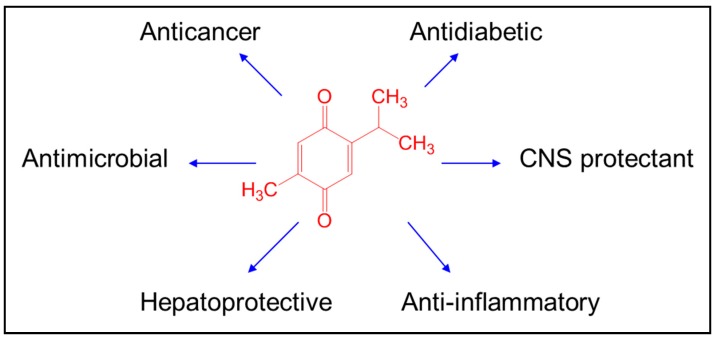
Biological activities of thymoquinone.

Botanical and natural drugs have a long, documented history in treating numerous diseases. *Nigella sativa* grows in the Mediterranean and in Western Asian areas. *N. sativa* is one of the most famous herbs used in Islamic traditional medicine. The *N. sativa* seeds have many pharmacological properties such as antioxidant, anticarcinogenic, antihypertensive, and antidiabetic [1,2,3]. Thymoquinone (TQ) is the main constituent of *N. sativa* and has powerful anticancer, antioxidant, antimicrobial, immunomodulatory, antihistaminic, and anti-inflammatory biological properties [4,5] (Figure 1). TQ has broad nutraceutical potential that includes anti-inflammatory, antioxidant, and anticancer uses, but its high instability [6], rapid elimination, and more than 99% binding to plasma proteins [7] limits the clinical outcome of TQ treatment [8]. Several elegant, detailed reviews covering the above aspects have been published [8,9,10,11,12].

**Figure 2:**
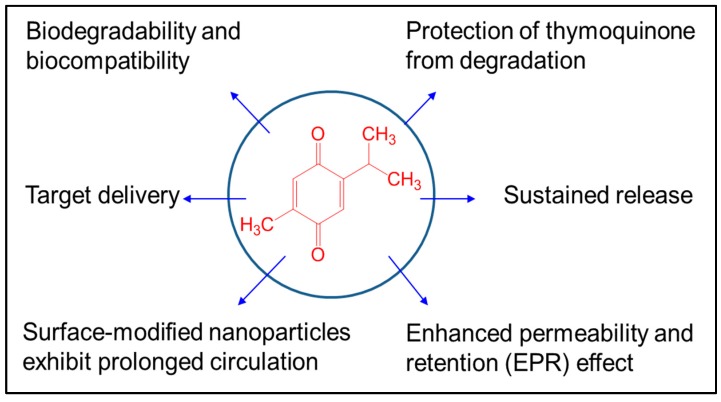
Advantages of thymoquinone nanoformulations.

**Figure 3:**
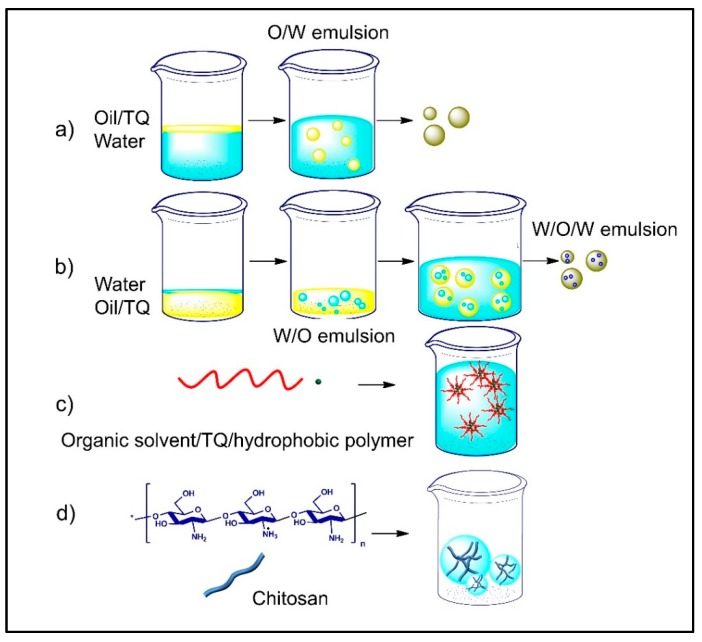
Thymoquinone nanoformulations prepared through (**a**) single emulsion; (**b**) double emulsion; (**c**) nanoprecipitation; (**d**) ionic gelation methods.

Nanonutraceuticals have promising properties that help to overcome the limitations of a drug that has a narrow therapeutic window or low bioavailability (Figure 2). Nanonutraceuticals provide protection and reduce renal clearance for a prolonged TQ pharmacological effect. As an ideal nanonutraceutical, TQ would be delivered to the target tissue or organ, where free TQ could then reach a therapeutic concentration and be maintained for a required time. Nanomaterials can easily penetrate the biological membranes and provide sustained release of TQ to different body parts [13], and therefore, nanoformulated TQ would enhance its bioavailability. Enhancement of drug and food constituents’ delivery and therapeutic effects have been achieved by a wide assortment of bottom-up encapsulation methods, such as single emulsions, double emulsions, nanoprecipitation, or the ionic gelation method [14], biopolymer side chains conjugations [15], and top-down methods such as the cold wet-milling method [16] (Table 1, Figure 3). In addition, nanoparticles (NPs) have been extensively used for drug delivery enhancement such as carbon, ceramic, and chitosan NPs [17].

Some studies have been done to enhance the bioavailability of TQ, especially the oral route, including micelle NPs, chitosan NPs, and liposomes [23,24,25]. Oral liquid formulations of TQ enhanced TQ’s solubility and bioavailability and protected it from photodegradation [6]. Nano-TQ has more photostability and a 6-fold increase of oral bioavailability than free TQ solution [16]. In addition, Tubesha et al. [26] indicated that TQ nanoemulsion is stable for 6 months. We conducted this review to throw light on the effect of TQ nanoformulations on the efficacy of TQ in control of human diseases. Finally, future directions for improving the pharmacokinetics of TQ and perspectives on the clinical translation of nano-TQ are discussed.

## 2. Biological Activities of Thymoquinone Nanoformulation

### 2.1. Anticancer

**Figure 4:**
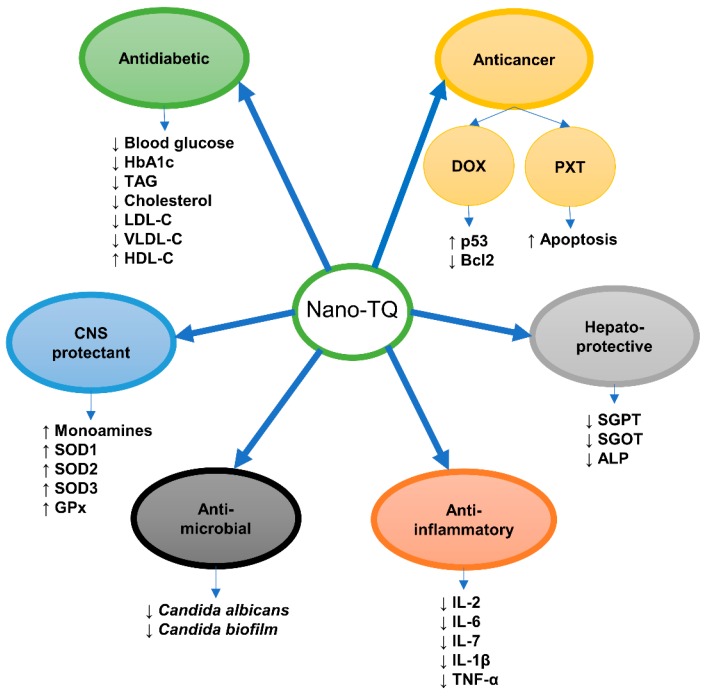
Biological activities of thymoquinone nanoformulations. ALP, alkaline phosphatase; Bcl2, B-cell lymphoma 2; DOX, doxorubicin; GPx, glutathione peroxidase; HbA1c, glycated hemoglobin; HDL-C, high-density lipoprotein-cholesterol; IL, interleukin; LDL-C, low-density lipoprotein-cholesterol; PTX, paclitaxel; SGOT, serum glutamate oxaloacetate transaminase; SGPT, serum glutamate pyruvate transaminase; SOD1, Copper, zinc-dependent superoxide dismutase (cytosolic); SOD2, manganese-dependent superoxide dismutase (mitochondrial); SOD3, Copper, zinc-dependent superoxide dismutase (extracellular); TAG, triacylglycerol; TNF-α, tumor necrosis factor-α; TQ, thymoquinone; VLDL-C, very low-density lipoprotein-cholesterol.

As concluded in our previous comprehensive review about the possible anticancer effect of TQ, TQ is a powerful anticancer component of *N. sativa* through its regulation of the diversity of cell signaling pathways and other cell components [2]. Nanoformulation of TQ potentiated its anticancer effect. Topotecan and TQ were loaded in poly(d,l-lactide-*co*-glycolide) (PLGA) nanomatrix where topotecan is dissolved in the inner aqueous phase while TQ is loaded into the organic phase of the double emulsion. This nanomatrix enables co-delivery of topotecan and TQ and leads to enhancement of the anticancer effect of topotecan-TQ formula [27] (Figure 4).

El-Ashmawy et al. [20] loaded doxorubicin (DOX) and TQ into F2 gel (fully acetylated poly-*N*-acetyl glucosamine nanofiber). The DOX-TQ combination exhibited significant reductions in tumor volume due to B-cell lymphoma 2 (Bcl2) downregulation and P53 upregulation compared to free conventional therapies that favor apoptosis, along with protection of the heart from DOX cardiotoxicity. DOX-TQ-loaded F2 gel showed a notable anticancer potential. This indicated that nanoformulation of DOX-TQ combination facilitates the drug delivery and enhances the anticancer effect of DOX with limited cardiotoxicity.

**Figure 5:**
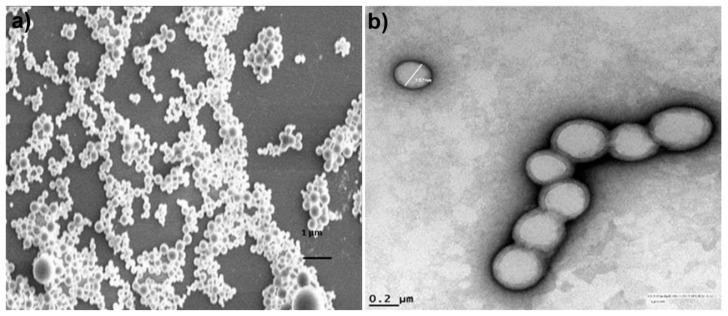
(**a**) Scanning electron microscope (SEM) and (**b**) transmission electron microscope (TEM) images of TQ/PLGA nanoparticles formulated with single emulsion solvent evaporation method. Reprinted with permission from Ref. [18].

PLGA NPs encapsulating paclitaxel (PTX) and/or TQ were formulated using a single emulsion solvent evaporation method (Figure 5). NPs that contained both PTX and TQ induced significant apoptosis in MCF-7 breast cancer cells compared to the free drugs, indicating the synergistic effect of both nano drugs. Therefore, PTX-TQ NPs exhibited an improved anticancer effect and can alleviate the PTX-associated toxicities [18]. 

To combat TQ’s low aqueous solubility, thermal properties, photosensitivity, and bioavailability, Bhattacharya et al. [21] synthesized TQ-encapsulated NPs using polyvinylpyrrolidone and polyethyleneglycol. These TQ-NPs showed more effectiveness in breast cancer cell apoptosis and had less toxicity to the normal cells compared to free TQ. These NPs effectively retarded breast cancer cells’ migration by downregulation of actin cytoskeleton that was initiated by upregulation of miR-34a. 

The anticancer effect of nano-TQ still need more investigations to determine its efficacy in comparison to conventional TQ and chemotherapeutic agents. Also, the effect of nano-TQ on different pro-apoptotic and anti-apoptotic pathways needs extensive work to determine the beneficial anticancer effect of TQ nanoformulations.

### 2.2. Antidiabetic

Diabetes is a metabolic disease mediated by disturbances in insulin secretion, action, or both is and associated with hyperglycemia, which leads to oxidative injuries of different organs [28]. 

TQ is a promising antidiabetic agent controlling the diabetic complications in different organs [29,30]. Rani et al. [22] investigated the antidiabetic benefit of nano-TQ in streptozotocin-nicotinamide induced type-2 diabetes in rats and compared them with metformin. The authors prepared nano-TQ and metformin by a nanoprecipitation with gum rosin and then characterized these nanoformulations before supplementation to diabetic rats for 21 successive days. These TQ- and metformin-loaded nanocapsules showed a sustained release profile as compared to their free forms. TQ, metformin, and their nanoformulations significantly decreased the blood glucose and glycated hemoglobin levels accompanied with improvement in the serum lipid profile of the diabetic rats. TQ-loaded nanocapsules overcame hyperglycemia in diabetic rats in a dose-dependent manner comparable with TQ and metformin. This study proved that nanoformulation of TQ enhanced its antidiabetic effect. Further molecular investigations are necessary to determine the molecular mechanisms of nano-TQ antidiabetic activity. This study is the single one concerning the antidiabetic effect of nano-TQ, but without determination of the molecular mechanism of nano-TQ action (Figure 4). We recommend that researchers determine the role of nano-TQ in pancreatic cell regeneration, insulin secretion, and insulin sensitivity.

### 2.3. Central Nervous System (CNS) Protectant

The CNS is continuously subjected to the hazards of different pollutants and some inflammatory diseases. TQ has been used as a natural protectant to various CNS diseases including glioblastoma, Alzheimer’s, and Parkinson’s diseases [31,32,33].

Alam et al. [34] studied the possible effect of TQ-loaded solid lipid nano particles (TQ-SLN) on the brain, comparing it to free TQ through the determination of a modified forced swim test, tail suspension test, and locomotor activities in rats. They found that TQ-SLN increased TQ delivery to brain tissues faster than free TQ, as demonstrated by determination of 5 hydroxytryptamine, dopamine, and norepinephrine levels in the brain (Figure 4). Briefly, TQ-SLN successfully improved TQ efficacy on brain tissues and may be of great importance for the treatment of different CNS diseases. Another study was done to compare TQ-rich fraction (TQRF) and TQ in nano- and conventional emulsions on rats fed with a high fat-cholesterol diet (HFCD). TQRF-nanoemulsion supplementation to HFCD fed rats ameliorated memory deficit and soluble β-amyloid levels besides enhancing the antioxidant status in brain cortex and hippocampus [35].

### 2.4. Anti-Inflammatory

Numerous studies have used conventional TQ as an anti-inflammatory agent in various animal models [30,36]. However, the poor aqueous solubility and photosensitivity of TQ hinders its bioavailability. Therefore, Jain et al. [37] prepared TQ lipospheres for topical use as an antipsoriatic drug. Lipospheres are a powerful means for drug delivery with stability and scalability. The authors used the murine macrophage cell line RAW 264.7 and they observed decreases in the levels of nitric oxide, interleukin-2 (IL-2), IL-6, IL-1β, and tumor necrosis factor-α (TNF-α), whereas in vivo (BALB/c mice), TQ lipospheres histopathological features reduced IL-17 and TNF-α in psoriatic skin with noticed ameliorations in histopathological features (Figure 4). Concomitantly, TQ lipospheres are a promising antipsoriatic drug and control inflammation. Nanoformulation increased the efficacy of TQ as a topical drug with more stability. 

This topic needs more research to elucidate the anti-inflammatory effect of nano-TQ in in vitro and various animal models either using lipopolysaccharides (LPS) or other chemically induced inflammatory process.

### 2.5. Hepatoprotective

Liver as an essential organ has an important function in metabolism and xenobiotic detoxifications. Oxidative stress is the main cause of hepatic injuries. The seeds of *N. sativa* are widely used as a hepatoprotective medicinal herb. In addition, TQ has hepatoprotective effects against injures through different mechanisms including mainly radical scavenging [38]. Many trials have been done to prepare TQ nanoformulations to enhance their hepatoprotection. Kalam et al. [39] developed different self-nanoemulsifying drug delivery systems (SNEDDS) formulations of TQ for in vivo hepatoprotective investigations in rat models. TQ-SNEDDS significantly protected the liver in comparison with TQ suspension due to improvement in TQ absorption and the relative bioavailability of TQ-SNEDDS. In the same manner, Elmowafy et al. [19] used a male rat model and enhanced the oral bioavailability of TQ by loading it in nanostructured lipid carriers. 

Singh et al. [40] formulated and characterized TQ-SLNs for treatment of liver cirrhosis. TQ-SLNs exhibited more stability than free TQ-suspension. The authors compared TQ-SLNs, free TQ, and SILYBON^®^ formulations for protection against liver cirrhosis induced by paracetamol, and they found that TQ-SLNs significantly decreased serum glutamate oxaloacetate transaminase (SGOT), serum glutamate pyruvate transaminase (SGPT), and alkaline phosphatase (ALP) (Figure 4).

The hepatoprotective effect of TQ is due to the potent antioxidant potential of TQ, while the nanoformulations potentiated the antioxidant power of TQ.

### 2.6. Antimicrobial

One of the most serious worldwide health problems and major challenges is the multidrug-resistant bacteria that has developed in treatment of infectious diseases [41]. Therefore, many medicinal plants are used as alternative antimicrobial drugs [1]. TQ displays a critical antibiotic role and may be able to decrease bacterial resistance to distinct antibacterial drugs [42].

One trial was done to explore the in vitro antifungal effect of amphotericin-B-, ketoconazole-, and TQ-NPs against *Candida albicans* yeasts and *Candida biofilm* in comparison to their conventional forms. *Candida* yeasts and *Candida biofilm* were two to four times more effectively disinfected by amphotericin-B-NPs (0.31 µg/mL), ketoconazole-NPs (0.62 µg/mL), and TQ-NPs (160 µg/mL) [43] (Figure 4). Further research is mandatory to determine the antifungal properties of TQ nanoformulations.

## 3. Applications of Nano-TQ

### 3.1. Nano-TQ Cosmetics

Liposomes were one of first nanoformulations to hit the market because they can keep the encapsulated nutrient safe from the environment and can improve the solubility of a hydrophobic nutrient in semi-solid or liquid form. In addition, they enhance the topical bioavailability because NPs can penetrate deeper into the skin [44,45,46]. The antioxidant and anti-apoptosis powers of TQ have been proven to create an anti-aging effect in mice [47]. TQ-SLN formulations have been prepared via high-pressure homogenization for anti-aging, moisturizing, and protective cosmetics. Most importantly, the high physical stability of nano-TQ at various storage temperatures has been demonstrated [48]. However, there are still safety concerns about the consequences of nano-TQ contact with human skin, and nano-TQ may trigger an inflammatory defensive reaction when used long-term [46,49]. 

### 3.2. Health and Nutritional Supplement Drinks of Nano-TQ

**Figure 6:**
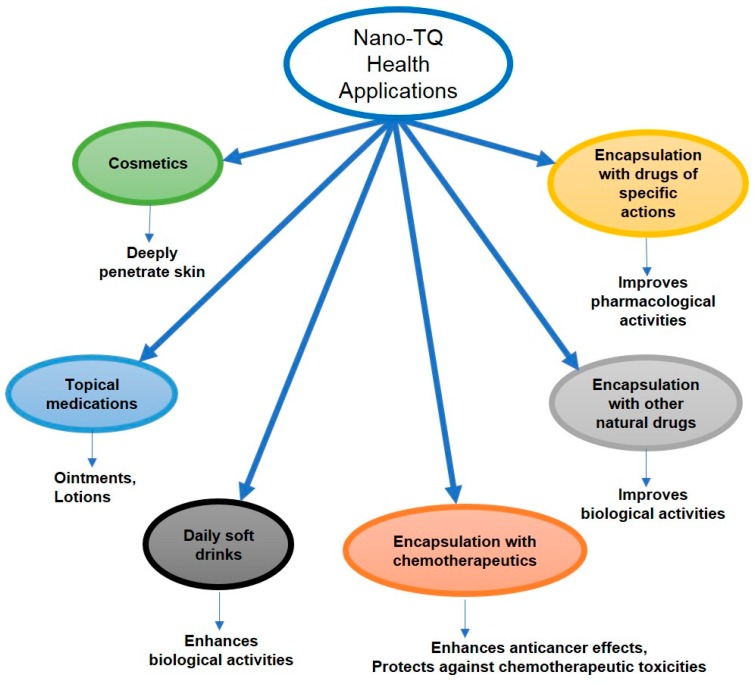
Nano-TQ and its uses in health and nutrition.

Many nutritional supplements are on the market that are formulated using nanotechnology, such as beta-carotene, lycopene, lutein, CoQ10, and omega 3 fatty acids. Nano-TQ also has shown higher oral bioavailability, as indicated in Table 1. TQ as a natural, biologically active compound has hepatoprotective, anti-inflammatory, antioxidant, and anticancer effects that encourage the use of nano-TQ as a health and nutritional supplement (Figure 6). Jazieh et al. reported that TQ was very commonly used in the Middle East by cancer patients as a nutritional supplement along with chemotherapy [50]. Nano-TQ as a nutritional supplement may augment the therapeutic effect and diminish the toxicity of chemotherapy drugs.

## 4. Overcoming the Roadblocks in Clinical Translation of Nano-TQ

Formulation of TQ provides a simple and low-cost method for producing water solubility and stability of TQ in a controllable manner and improves the efficient transport of TQ across the biological barriers. However, clinical translation of NP-based TQ remains challenging. The key to success in nano-TQ delivery systems is to solve the formulation problems at the beginning. That means that a prodrug strategy for TQ should be considered to improve the pharmacological potentials of TQ. It may be possible for synthetic chemists and computer scientists to modify the structure of TQ to develop more drug-likeness of TQ for encapsulation within nanoformulation. For example, absorption, distribution, metabolism, and excretion (ADME) in silico modelling such as ADMET Predictor (Simulations Plus, Inc., Lancaster, CA, USA), GastroPlus (Simulations Plus), and Derek and Meteor (Lhasa Limited, Leeds, UK) can be used to predict ADME information and drug metabolism and pharmacokinetics (DMPK) data for designing promising prodrugs [51].

The excipient used in the formulation of nano-TQ delivery systems should have a high degree of biocompatibility, biodegradability, and simple composition. Nano-TQ formulations usually contain a large amount of excipient, and therefore, the excipient should not have side effects. For example, Cremophor-EL is used in the conventional formulation of Taxol for increasing the solubility of the hydrophobic drug PTX. However, Cremophor-EL has toxicity issues such as lethal hypersensitivity and anaphylactic reactions. To overcome these issues, formulation scientists should select safer nanocarriers, such as endosomes and biosurfactants that are biodegradable and have good biocompatibility [52,53]. 

Nano-TQ can effectively deliver enough TQ to target sites of disease. In other words, high TQ loading in nano-TQ and specific transport pathways are key for nano-TQ to be successful. For example, the first FDA-approved nanomedicine, PEGylated liposomal DOX, has >90% DOX encapsulation and around 10% DOX loading. Remote loading of DOX, driven by the transmembrane ammonium sulfate gradient, was the main reason for the success of Doxil^®^ (Janssen Products, LP, Horsham, PA, USA) [54]. Indeed, PEGylated liposomal DOX demonstrates a prolonged half-life of DOX, however, a high concentration of DOX in plasma and skin leads to a side effect called hand–foot syndrome. The first protein-based nanomedicine, nab-paclitaxel, has 10% PTX loading, and particularly important is the fact that nab-paclitaxel can penetrate tumor extracellular matrix through caveolar transport pathways and gp60 albumin receptor. The nab-paclitaxel formulation is simple and effective [55]. Based on the ability of exosomes to easily fuse with cell membranes and deliver drugs directly into the cytosol along with a high number of recent literature citations on endosomal delivery strategies, endosome-based nanomedicines may reach the market soon [56]. 

TQ and excipient compatibility should be determined as a primary screening step. Chemical compatibility should be tested with high-performance liquid chromatography (HPLC) and nuclear magnetic resonance spectroscopy (NMR); physical compatibility should be tested with dynamic light scattering (DLS), transmission electron microscope (TEM), scanning electron microscope (SEM), and X-ray powder diffraction (XRD). For further investigation, a small-angle neutron scattering study could help to understand in situ formation of NPs in aqueous solution, and 2D TOCSY NMR could be used to study the interaction between API and delivery systems. For example, 2D- TOCSY NMR could be a useful analytical technique for TQ and β-cyclodextrins, poly (vinyl alcohol) and poloxamers in complex [57].

The formulation of nano-TQ delivery systems is usually a multistep process. At small scale, it is easy, but at the larger scale, or even industrial scale, it becomes more difficult to control the batch-to-batch reproducibility. To overcome this, cold wet milling, spray drying, and a microfluidics system have been developed for efficient clinical translation of nanomedicines [58]. Wet milling and several microfluidics-based instrument for supporting the scale-up or GMP manufacturing have hit the market, such as High Energy Ball Mill Emax (Retsch, Castleford, UK), Microfluidizer^®^ (Microfluidics Corp., Westwood, MA, USA), NanoAssemblr^™^ (Precision NanoSystems, South San Francisco, CA, USA), MicroJet Reactor (MJR^®^) (leon-nanodrugs Inc., New York, NY, USA), and Nano Spray Dryer B-90 (BUCHI Co., New Castle, DE, USA) (Table 2).

Finally, TQ’s low aqueous stability may be a challenging issue in the nanomanufacturing process. It may affect the purification, lyophilization, and/or sterilization process of nano-TQ. To limit a clinical translation gap, strong collaborations between academic labs and pharmaceutical companies need to be formed.

## 5. Conclusions

Nanoformulations of TQ potentiate the pharmacological potentials of TQ via enhancement of TQ’s bioavailability with significant decreases in its required doses. We strongly recommend more investigation on nano-TQ’s efficacy in different diseases and their application in human disease prevention through incorporation of nano-TQ in drug formulations, nano cosmetics, and several health and nutritional drinks.

## Figures and Tables

**Table 1:** TQ and TQ nanoformulations and characteristics.

Method	Materials, Stabilizer	Size (nm)	Animal, Dose	Therapeutic Effect	Reference
Single emulsion	PLGA	200~300		Enhanced anticancer activity	[18]
	Compritol ATO 888, gelucire	~200	Rats, 20 mg/kg, oral	4-Fold enhancement of oral bioavailability	[19]
Double emulsion	Poly-*N*-acetyl glucosamine, PVA	185	Mice, ~150 mg/kg, subcutaneous injection	Inhibited tumor growth 43%, 31%	[20]
Nano-precipitate	PVP, PEG_200_, PEG_4000_, P123	20~40	Mice, 5 mg/kg, subcutaneous injection	Reduced tumor and increased lifespan	[21]
	Gum rosin, oleic acid, PVA, polysorbate 80	50–90	Wistar female albino rats, 20, 40, 80 mg/kg, oral	Significantly decreased blood glucose level and glycated hemoglobin	[22]
Ionic gelation	Chitosan, TPP	150	Male Wistar rats, 2.52 mg/kg, intranasal	15-Fold higher brain targeting efficiency	[23]
Film rehydration	Liposomes	100		More potent anti-proliferative activity	[24]
Cold wet-milling	HPC-SSL	143	Male Sprague-Dawley rats, 2 mg/kg, oral	6-Fold enhancement of oral bioavailability	[16]

Abbreviations: PLGA, poly(d,l-lactide-*co*-glycolide); PVA, poly(vinyl alcohol); PVP, polyvinylpyrrolidone; PEG, poly(ethylene glycol); P123, poly(ethylene glycol)-*b*-poly(propylene glycol)-*b*-poly(ethylene glycol); TPP, sodium triphosphate; HPC-SSL, hydroxypropyl cellulose grade SSL.

**Table 2:** Critical parameters for scale-up or GMP manufacturing of nanomedicine technology for nano-TQ.

	Thermal Stress	Complexity	Scale-Up Principle	Organic Solvent
Ball Mill Emax (wet milling)	Yes	>50 cycles	Top-down	No
Microfluidizer^®^	No	5–6 cycles	Bottom-up (single emulsion)	Yes
NanoAssemblr™	No	1-step	Bottom-up (nano-precipitate)	Yes
MicroJet Reactor (MJR^®^)	No	1-step	Bottom-up (nano-precipitate)	Yes
Nano Spray Dryer B-90 (spray-drying)	No	1-step	Bottom-up (freeze-dry)	Yes
